# Image Analysis and Data Normalization Procedures are Crucial for Microarray Analyses

**DOI:** 10.4137/grsb.s414

**Published:** 2008-03-17

**Authors:** Ali Kpatcha Kadanga, Christine Leroux, Muriel Bonnet, Stéphanie Chauvet, Bruno Meunier, Isabelle Cassar-Malek, Jean-François Hocquette

**Affiliations:** INRA, UR1213, Unité de Recherches sur les Herbivores, Centre de Recherches de Clermont-Ferrand/Theix, F-63122 Saint Genès-Champanelle, France

**Keywords:** microarray, bovine, data analysis, experimental design, statistical analyses

## Abstract

This study was conducted with the aim of optimizing the experimental design of array experiments. We compared two image analysis and normalization procedures prior to data analysis using two experimental designs. For this, RNA samples from Charolais steers *Longissimus thoracis* muscle and subcutaneous adipose tissues were labeled and hybridized to a bovine 8,400 oligochip either in triplicate or in a dye-swap design. Image analysis and normalization were processed by either *GenePix/MadScan* or *ImaGene/GeneSight*. Statistical data analysis was then run using either the SAM method or a Student’s *t-*test using a multiple test correction run on R 2.1 software. Our results show that image analysis and normalization procedure had an impact whereas the statistical methods much less influenced the outcome of differentially expressed genes. Image analysis and data normalization are thus an important aspect of microarray experiments, having a potentially significant impact on downstream analyses such as the identification of differentially expressed genes. This study provides indications on the choice of raw data preprocessing in microarray technology.

## Introduction

Microarray technology is gaining ground as an approach for exploring major subsets or almost complete gene profiles of organisms. The technology makes it possible to analyze a variety of conditions such as samples of several treatments, mutants, developmental stages or time points. However, gene expression values derived from microarrays are often considered less reliable than other biochemical data (Mangalathu et al. 2001). Experimental biases in gene expression profiling could occur due to varying total amounts of hybridized mRNA, different label incorporation rates, the methodology applied for spot quantification trough image analysis, or bleaching effects of the dye. The comparability and reliability of data generated using microarray technology could be enhanced by using of a common set of standards, as recently proposed (Shi et al. 2006), which would allow better accuracy and reproducibility as well as dynamic range assessments, although probably not without certain persistent problems.

Variability in microarray experiments can arise from either pre-scanning steps (methods of RNA extraction, different types of probe preparation, probe labeling, hybridization and slide quality) and/or post-scanning steps (image acquisition and image/data analysis), as previously reported (Ahmed et al. 2004). Relatively little attention has been given to the variability introduced by image analysis methods as a potential source of noise. Variability introduced by image analysis may predominantly be generated by the method used to estimate signal background from a spot and segmentation. Another important point to emphasize is that the one-color and two-color designs yield equivalent data, yet this variable is not considered as a critical factor (Patterson et al. 2006).

Normalization methods have been developed for assessing spot quality in an extended examination factoring in spot size, signal-to-noise ratios, background uniformity, and saturation status. For segmentation methods, examples of algorithm and software implementations have been described elsewhere (Yang et al. 2001), and comparisons have been performed between the algorithms used by different segmentation methods (Yang et al. 2000). It has been shown that small and large-scale fluctuations in pixel intensities within a spot lead to uncertainty in microarray quantitation, and that pixel-to-pixel variability correlates with variability between replicate spots on duplicate slides (Brown et al. 2001). To underline these issues, most investigators use the commercial software provided (for instance *GenePix*, *ImaGene*, *QuantArray* or *ScanAnalyze*) without further development of the methods.

The aim of the statistical tests (Student’s *t-*test, significance analysis of microarrays (SAM)) is to estimate the significance of differential gene expression. However, the statistical power of these tests depends on a sufficient number of repeated hybridizations. Every major platform provider or laboratory has its own preferred algorithm for array data analysis, but there is still no consensus on the best way to analyze microarray data. It has been demonstrated that different methods of analysis can result in a very different outcomes from the same dataset (Harr and Schlötterer, 2006).

The aim of the research reported here was to assess different microarray procedure, based on different image analysis and statistic methods by focusing on variability in the results. We have analyzed two different datasets (obtained after hybridization of the same samples on the same slides with two experimental designs) using two image analysis/normalization methods and two statistical approaches. We show that using software such as *GenePix/MadScan* or *ImaGene/GeneSight* may influence the outcome of differentially expressed genes, regardless of the statistical method used.

## Materials and Methods

### Biological samples

For the present study, we used *Longissimus thoracis* (LT) muscle and subcutaneous (SC) adipose tissue samples from 30-month-old Charolais steers. The samples were excised within 10 min. postmortem and immediately frozen in liquid nitrogen and stored at −80 °C until analysis.

### RNA isolation and labeling

Total RNA was extracted from SC adipose tissue as described previously (Bonnet et al. 1998) and extracted from LT muscle using TRIzol Reagent (Life Technologies, UK). The total RNA was then purified and treated with DNAseI using the RNeasy^®^ Mini kit (Qiagen, France) as recommended by the manufacturer. RNA integrity was checked using Lab Chip Agilent technology, as previously described (Bernard et al. 2007). The reference corresponded to a mixture of same quantities of total RNA from SC, LT and mammary gland samples. Mammary gland RNA was used as a positive control since 6 genes are highly expressed in this organ.

### Microarray hybridization and data acquisition

**Figure 1:**
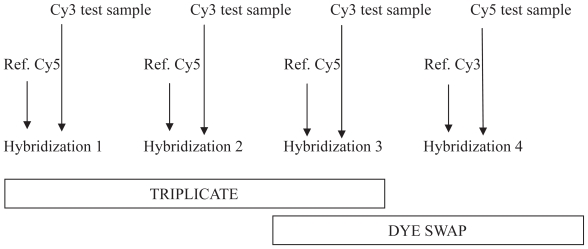
Experimental design used in this study. Test sample is RNA of the target sample (LT and SC) to be studied and Ref. is the reference (RNA from LT, SC and mammary gland).

Labeling was performed using a “Pronto Plus Direct Systems” kit (Corning-Promega Europe, France) according to the supplier’s guidelines. Five μg of total RNA was reverse transcribed into cDNA in the presence of cyanine (Cy3-dCTP/Cy5-dCTP, Amersham, UK). cDNA purification was performed according to the manufacturer’s instructions, and concentration and frequency of incorporation were determined by spectrophotometry. Cyanine 3 and cyanine 5-labelled cDNA (40 pmol each) were mixed, dried and resuspended in 40 μl “long-oligo” buffer before hybridization onto one microarray (8400 probes from Operon, Corning Ultra GAP-spotted in Genopole Toulouse, France). Hybridization was performed at 42 °C for 16 h as previously described (Ollier et al. 2007). Post-hybridization washes followed by slide centrifugation were carried out before the scan. The experimental design (Fig. 1) was as follows for each sample (hybridization 3 from triplicate was used as dye swap in the data analysis). Hybridized microarrays were scanned with a 428 MWG Array Scanner (MWG Biotechnologies).

### Data analysis

Image analysis was achieved using two different software solutions: *GenePix* Pro 6.0 (Axon Instruments, USA) and *ImaGene* 6.0 (BioDiscovery, USA). The rationale for using these programs was that they are based on different extracting (or segmentation) methods, especially for the determination of background. GenePix uses fixed circle and adaptive shape with background, with median from “valley of spots” algorithms, while *ImaGene* is based on non-fixed spotting using Mann-Whitney test algorithms.

Raw data were filtered and normalized using either *MadScan* 6.0 (http://cardioserve.nantes.inserm.fr/mad/madscan) or *GeneSight* 4.1.6 (BioDiscovery, USA) software. MadScan proceeds by filtration, the Lowess fitness method (within-slide) plus scaling, outlier detection, and finally significantly expressed genes detection (between slides) in a single or multiple analysis step (Le Meur et al. 2004), whereas *GeneSight* (http://www.biodiscovery.com/index/gen-esight) uses the global lowess fitness method after the replicates have been combined.

Hybridization data were run through SAM (Significance Analysis of Microarray) analysis (Tusher et al. 2001) or multi-test model. The last statistical analysis was performed after filtering using R 2.1 software: after controlling the variance of each gene, log of ratios between Sample and Reference were analyzed with an ANOVA model by using standard Student’s *t* test to detect differentially expressed genes. Probability values were adjusted using the Bonferroni correction for multiple testing at 1% level to eliminate false positives. We have named this last procedure: *Multi-test*.

Control and common genes were determined between the results from *GenePix/MadScan/SAM* and *ImaGene/GeneSight/SAM* to make it possible to compare the image analysis and normalization procedures. For instance, the statistical method effects could be assessed by comparing data processed by ImaGene/GeneSight/SAM and ImaGene/GeneSight/Multi-test. The common genes between GenePix/*MadScan/SAM* and *ImaGene/GeneSight/Multi-test* were used to assess the combined effects of image analysis, normalization and statistical methods.

## Results

The number of differentially expressed genes with each procedure is summarized in Table 1. Globally for high fold change (FC > 2), both the combination of image analyses and normalization using *GenePix/MadScan* or *ImaGene/GeneSight* gave a fairly similar number of genes declared statistically differently expressed, whereas for low level (FC > 1.4), there were clear differences in gene numbers. The following interpretation of the results was referred to the lower fold change (FC > 1.4) in triplicate experimental design.

To have positive control, we have implemented the reference sample with mammary RNA. Milk proteins genes (CSN1S1, CSN1S2, CSN2, CSN3, LALBA and LGB) were specially expressed in the mammary gland of lactating cow. Thus, compared to LT or SC tissues, these genes could be used as positive control of differentially expressed genes. We observed that the differential expression on these 6 genes depended on the combination of the image analysis system with the normalization procedure used.

Within the triplicate experimental design, only one gene (CSN2, FC = 5.4) was declared differentially expressed with the *GenePix/MadScan* combination, versus six with *ImaGene/GeneSight* (regardless of statistical approach) in LT sample. The same gene (CSN2, FC = 9) was declared differentially expressed with the *GenePix/MadScan* combination versus 5 (CSN1S1, CSN2, CSN3, LALBA and LGB) with *ImaGene/Gensight* in SC sample whatever the statistical analysis.

### Comparison of two image analysis/normalization procedures

Under the triplicate design and with the same statistical procedure (SAM), the number of genes that exhibited statistically significant changes (p < 0.001) in expression (FC > 1.4) was 352 for the *GenePix/MadScan* combination and 140 for the *ImaGene/GeneSight* combination with 104 common genes (52%) in LT (in part a of the table). In SC sample, 384 versus 216 genes were observed in *GenePix/MadScan* and *ImaGene/GeneSight* combinations respectively with 142 common genes corresponding to a proportion of 51%.

### Comparison of two statistical methods

Statistical analysis was evaluated under the triplicate experimental design, after image analysis and normalization procedures by *ImaGene/GeneSight* (in part b of the table). The data analyzed by both statistical procedures (SAM and Multi-test with FC > 1.4) led to a nearly comparable number of genes declared as differentially expressed. In LT sample, 129 genes (77%) were common to 140 genes from *ImaGene/GeneSight/SAM* and 207 genes from *ImaGene/GeneSight/Multi-test*. Similar proportion (72%) of genes common (121 in number) in SC sample (216 in *ImaGene/GeneSight/SAM* versus 138 in *ImaGene/GeneSight/Multi-test*) was observed.

### Comparison of two experimental designs

Finally, we have compared triplicate and dye-swap experimental design using the same process of data (*ImaGene/GeneSight/Multi-test*: part c of the table). The comparison of results obtained with these two experimental designs showed similar number of genes identified, in particular in LT tissue. Regarding the positive control, the same genes were highlighted. Thus, in SC, 6 *versus* 5 genes (CSN1S2, CSN2, CSN3, LALBA and LGB) were detected.

## Discussion

The purpose of this study was to compare two post-scanning methods (*ImaGene/GeneSight* and *GenePix/MadScan*), two different statistical methods (SAM and Student’s *t*-test) and two experimental designs (triplicate and dye swap) for analyzing microarray data. Our results show that the highest differences between procedures were obtained with FC > 1.4 rather than FC > 2. This was observed in terms of number of genes declared as statistically differently expressed and number of common genes.

### Importance of image analysis and data normalization

The comparison of two post-scanning methods prior to statistical analysis (*ImaGene/GeneSight* and *GenePix/MadScan*) using some dataset showed that treatment with *ImaGene/GeneSight* outlines more consistence genes than *GenePix/MadScan*, in particular regarding the 6 positive control genes introduced into our experience. The presence of 6 of these genes was considered as a test of results reliability in this study. We observed differences in number of differential genes between procedures and two hypotheses could be made to explain these observations: *GenePix/MadScan* may induce false positives or *GenePix/MadScan* may be more efficient in detecting expression differences (fewer false negatives). The latest has been previously reported (Ahmed et al. 2004). The difference in the segmentation procedures between *GenePix* and *ImaGene* software (Yang et al. 2001; Ahmed et al. 2004) most probably explains the present results.

The combination of image analysis and data normalization is indeed an important aspect of microar-ray experiment. These steps can have a potentially large impact on downstream analyses such as the identification of differentially expressed genes. A number of microarray image analysis packages (commercial software and freeware) are now available. GenePix works along a principle based on fixed-circle and adaptive circle methods whereas *ImaGene* is based on histogram segmentation method, this last method being more powerful (Ahmed et al. 2004). The choice of the procedure used for these steps depends on the objectives. In our study, the procedure combining *GenePix/MadScan* was more appropriated to an explorative study whereas *ImaGene/GeneSight* was a good combination to identify candidate genes in relation with phenotype traits. A further advantage of *ImaGene/GeneSight* was the option of being able to confirm (or test) the yield of genes with different statistical methods such as SAM and the Student’s *t-*test.

### Importance of statistics

Designing procedures to control the FDR (criterion for identifying differentially expressed genes) are challenging problems (Verducci et al. 2006). SAM and multiple t-tests use R and its Bioconductor package. Before SAM can be run, there are some permutations that are needed to generate FDR. The Bonferroni, is a Family-Wise type Error Rate (FWER) method to correct for multiple testing to eliminate false positives (Lin, 2005). We aimed to compare the widely used SAM and the multi-test with p-value adjusted using Bonferroni correction, since there is no reference method to control the false positives. Based on our results, there was no significant statistical treatment-related difference in the number of differential genes.

### Importance of the experimental design

First, for the two samples studied, we proposed a “reference design” including an experimental procedure based on replicate spots within each micro-array. We compared hybridization to a reference sample with either a triplicate or a dye-swap design, as shown in Figure 1. This procedure allows us to minimize biological noise by reducing individual variability through testing sample-specific dye bias. We did not identify any significant difference between triplicate and dye swap, which is consistent with a previous study (Dobbin et al. 2005) reporting that sample-specific dye biases appeared to have only minimal impact on estimated gene-expression differences. However, we found that dye swap sorted some genes than triplicate design, although from limited samples, thus showing a gain in cost with no loss in efficiency (Dobbin et al. 2003).

## Conclusion

There are many sources of variability that can affect gene expression intensity measurements, but not all are well characterized or clearly identified. With these results, we demonstrated the impact of conducting the data extraction step before applying statistical models. From the above discussion, it is obvious why there can be a great deal of discordance in results obtained equally as often from different microarrays as from within the same microarray platforms. Ultimately, the decision between triplicate or dye-swap, software image analysis and normalization approaches is more often driven by cost, experimental design considerations, and objectives of the study. This raises the question of how array data can be compared. By presenting the experimental design and performance advantages of both models, we have provided insight and guidance for properly selecting the best approach depending of the objectives of the study.

## Figures and Tables

**Table 1:**
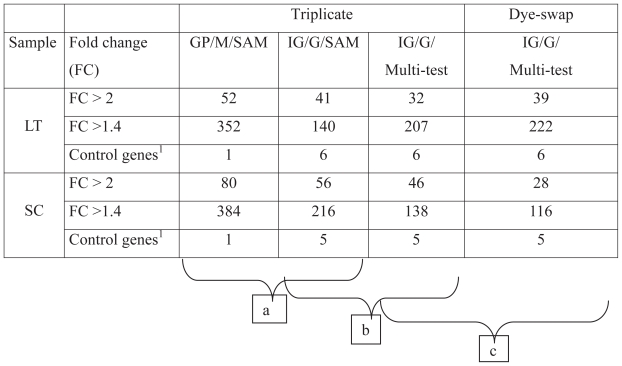
Number of differentially expressed genes after treatment of the same datasets from muscle (LT) or adipose tissue (SC) samples by GenePix (GP), MadScan (M), ImaGene (IG), GeneSight (G), SAM and Multi-test at different fold-change thresholds (FC).

**Abbreviations:** GP/M/SAM: GenePix/MadScan/Significance Analysis of Microarray; IG/G/SAM: ImaGene/GeneSight/Significance Analysis of Microarray; IG/G/Multi-test: ImaGene/GeneSight/Multi-test.

1 = control genes are the same for both fold change thresholds.

## Abbreviations

GP, M: GenePix, MadScan
IG, G: ImaGene, GeneSight
SAM: Significance Analysis of Microarray
LT: Longissimus thoracis muscle
SC: subcutaneous adipose tissue
CSN1S1: casein alpha s1
CSN1S2: casein alpha s2
CSN2: casein beta
CSN3: casein kappa
LALBA: lactalbumin alpha
LGB: lactoglubulin beta
FWER: family-wise type error rate
FDR: false discovery rate
FC: fold change
